# Association of leukocyte telomere length with risk of all-cause and cardiovascular mortality in middle-aged and older individuals without cardiovascular disease: a prospective cohort study of NHANES 1999–2002

**DOI:** 10.1007/s40520-024-02773-z

**Published:** 2024-06-13

**Authors:** Qianhui Wang, Linqiang Xi, Na Yang, Jie Song, Dilare Taiwaikul, Xiaoxue Zhang, Yakun Bo, Baopeng Tang, Xianhui Zhou

**Affiliations:** Department of Cardiac Pacing and Electrophysiology, The First Affiliated Hospital of Xinjiang Medical University, No. 137, Liyushan Road, Urumqi, 830054 PR China

**Keywords:** Telomere length, Mortality, NHANES, Biomarker

## Abstract

**Background:**

Leukocyte telomere length (LTL) shorting was significantly associated with mortality. This study aimed to investigate the potential association between LTL and all-cause mortality as well as cardiovascular disease (CVD) mortality in middle-aged or older individuals without a history of CVD.

**Methods:**

A total of 4174 participants from the National Health and Nutrition Examination Survey (NHANES) conducted between 1999 and 2002 were included in this analysis. Cox proportional hazards regression models were utilized to estimate the association between LTL and mortality outcomes. Restricted cubic spline (RCS) curves were employed to evaluate the potential non-linear association.

**Results:**

Over a median follow-up period of 217 months, the weighted rates of all-cause mortality and CVD mortality were 28.58% and 8.32% respectively. Participants in the highest LTL group exhibited a significantly decreased risk of both all-cause mortality (HR: 0.65, 95% CI: 0.54–0.78, *P* < 0.001) and CVD mortality (HR: 0.64, 95% CI: 0.45–0.93, *P* < 0.001) compared to those in the lowest group. Kaplan-Meier survival curves further supported a significant association between shorter telomere length and increased risks of both all-cause and CVD mortality (log-rank test *P* < 0.001). RCS curves demonstrated a linear dose-response relationship between LTL and all-cause mortality as well as CVD mortality. Subgroup and sensitivity analyses confirmed the robustness of the results.

**Conclusion:**

Shorter leukocyte telomere length could serve as a potential biomarker for risk stratification of all-cause and CVD mortality among middle-aged and older individuals without a history of CVD.

## Introduction

Telomeres, which are repetitive DNA sequences (TTAGGG) located at the ends of chromosomes, play a crucial role in maintaining genomic stability. During normal mitotic DNA replication, telomeric DNA undergoes shortening [1]. Once telomeres reach a critically shortened length, cells enter a state of senescence and lose their ability to divide further. Thus, telomere length (TL) has emerged as an important biomarker for biological aging [2, 3].

TL naturally undergoes significant shortening with advanced age [4]. However, factors such as chronic inflammation [5], oxidative stress [6], smoking, and obesity [7] also contribute to accelerated telomere shortening. Numerous studies have demonstrated that shorter leukocyte telomere length (LTL) is associated with an increased risk of age-related diseases, such as hypertension, diabetes, ischemic heart disease (IHD), and the corresponded mortality [8]. In fact, a prospective study conducted in Denmark’s general population revealed a significant positive association between shorter LTL and mortality [9]. However, some studies have contradicted these findings by suggesting that LTL is not a significant risk factor for the development of cardiovascular disease (CVD) [10, 11]. More recently, Chen et al. discovered a positive association between longer LTL and increased risk of CVD mortality among patients with type-2 diabetes in the United States (US) [12]. Consequently, the role of TL in the development of age-related diseases and long-term prognosis remains controversial.

To date, the prognostic value of LTL for long-term all-cause mortality and CVD mortality among middle-aged and older individuals without pre-existing CVD in the American population remains unclear. Hence, this study aims to investigate the association between LTL and long-term mortality outcomes in this specific population.

## Methods

### Study design and population

This study extracted data from the National Health and Nutrition Examination Survey (NHANES) cycles of 1999–2000 and 2001–2002, employing a prospective cohort design. The NHANES is an ongoing project conducted by the Centers for Disease Control and Prevention (CDC) to evaluate the health and nutritional status of individuals across all age groups in the United States. The study focused on individuals aged 40 years or older who underwent the LTL test, had follow-up data, did not have pre-existing cardiovascular disease (CVD), and had complete covariate data. The NHANES survey protocol underwent rigorous review and was approved by the research Ethics Review Board of the National Center for Health Statistics. All participants provided informed written consent.

### CVD diagnosis

Self-reported physician diagnoses obtained during individual interviews using a standardized medical condition questionnaire established the diagnosis of CVD. Participants were asked if they had ever received a diagnosis of congestive heart failure (CHF), coronary heart disease (CHD), angina pectoris, myocardial infarction (MI), or stroke from a doctor or other healthcare professional. Participants who answered positively to any of these conditions were classified as having CVD.

### Covariate data collection

Standardized questionnaires and laboratory tests were used to collect various covariate data. Demographic data, including age, gender, race/ethnicity, smoking status (never, former, and current), education levels, and family income, were recorded. Body mass index (BMI) was calculated as weight (kg)/height^2 (m). Blood pressure (BP) measurements were taken at the mobile examination center (MEC), and the average of three consecutive readings was calculated. Clinical indicators such as serum fasting glucose, HbA1c, triglycerides (TG), total cholesterol (TC), high-density lipoprotein cholesterol (HDL-C), uric acid (UA), and creatinine were analyzed in the NHANES laboratory. The estimated glomerular filtration rate (eGFR) was calculated using the CKD-EPI equation developed by the Chronic Kidney Disease Epidemiology Collaboration, which estimates renal function based on variables such as age, serum creatinine levels, gender, and race [13]. Hypertension was defined based on criteria such as physician diagnosis, use of antihypertensive medication, systolic BP (SBP) ≥ 140 mmHg, or diastolic BP (DBP) ≥ 90 mmHg [14]. Diabetes was classified if individuals met at least one of the following conditions: self-reported medical diagnosis of diabetes, fasting glucose ≥ 7.0 mmol/L, use of oral medications or insulin for glycemic control, or HbA1c ≥ 6.5% [15].

### LTL test

Blood samples were collected from participants in the NHANES survey, and the measurement of TL was performed using the quantitative polymerase chain reaction (PCR) method. The TL assay was conducted in the laboratory of Dr. Elizabeth Blackburn at the University of California, San Francisco. Each sample was assayed three times on three different days to ensure accuracy and reliability of the measurements. The assay involved duplicate wells for each sample, resulting in a total of six data points. To maintain quality control, the samples were assayed in groups of three plates, with no two plates being grouped together more than once. Each assay plate included 96 control wells containing eight control DNA samples. Any assay runs with eight or more invalid control wells were excluded from further analysis, which accounted for less than 1% of the runs. The control DNA values were used to normalize between-run variability. Furthermore, assay runs with more than four control DNA values falling outside 2.5 standard deviations from the mean for all assay runs were excluded from further analysis. This accounted for less than 6% of the runs. For each individual sample, potential outliers were identified and excluded from the calculations, which accounted for less than 2% of the total samples. Subsequently, the mean and standard deviation of the T/S ratio (telomere-to-single copy gene ratio) were calculated as usual. The inter-assay coefficient of variation was determined to be 6.5%.

### All-cause and CVD mortality ascertainment

The National Health and Nutrition Examination Survey public-use linked mortality file, as of December 31, 2019, was used to determine the mortality status in the population under follow-up. This file was connected to the National Death Index (NDI) through a probability matching algorithm conducted by the National Center for Health Statistics (NCHS). Additionally, we employed the International Statistical Classification of Diseases, 10th Revision (ICD-10), to identify deaths related to cardiovascular disease (054–068).

### Statistical analysis

During data analysis, the analytical guidelines provided by the National Center for Health Statistics were utilized, and sampling weights were considered due to the complex multi-stage sampling design of the NHANES. Participants were categorized into three groups based on the tertiles of LTL (T1: ≤0.882, T2: 0.882–1.071, T3: ≥1.071). Continuous variables were presented as mean ± standard deviation (SD) or median (interquartile range, IQR), depending on the distribution of the data. Differences between subgroups were evaluated using weighted one-way ANOVA or Kruskal-Wallis rank tests. Categorical variables were expressed as percentages (%) and analyzed using weighted chi-square tests.

To address the non-normal distribution, a natural logarithm (ln) transformation was applied to the t/s ratio. Weighted Cox proportional hazards regression models were employed to assess the association between LTL and both all-cause and CVD mortality. Model I was an unadjusted model, Model II adjusted for age, gender, race, education levels, smoking status, and BMI. Model III further adjusted for hypertension, diabetes, SBP, DBP, TG, TC, HDL, UA, BUN, and e-GFR. Kaplan-Meier curves were generated to visually depict the cumulative mortality risk among different groups and were tested using the log-rank test. Restricted cubic splines (RCS) were utilized to examine the potential non-linear dose-response relationship between LTL and the risk of mortality. Subgroup and sensitivity analyses were conducted to assess the robustness of the association.

Data analysis was performed using R studio (version 4.0.3) and Stata (version 14.0). Statistical significance was defined as *P* < 0.05.

## Results

### Baseline characteristics of participants according to the tertiles of LTL

A total of 4174 middle-aged or older participants without CVD were included in this study. Of these individuals, 2078 (49.78%) were male, and the average age was 58.99 ± 13.11 years. When comparing participants in the shortest group of LTL (T1) to those in the longest group (T3), several notable differences were observed. Participants in the T3 group tended to be younger, more likely to be female, have a higher proportion of non-smokers, and have achieved a college education or higher. Additionally, they had a lower proportion of hypertension and diabetes, lower levels of SBP, TG, BUN, and UA, as well as higher levels of DBP and eGFR (Table 1).

**Table 1:** Baseline characteristics of patients according to the tertiles of the leukocyte telomere length

Variables	T1 (*n* = 1391)	T2 (*n* = 1391)	T3 (*n* = 1392)	*P*-value
Age (years)	60.51 ± 12.79	54.88 ± 11.54	51.79 ± 10.13	< 0.001
BMI (kg/m^2^)	28.44 ± 5.74	28.59 ± 6.56	28.08 ± 5.93	0.327
Male (%)	49.49	47.64	45.37	< 0.001
Smoking status (%)				< 0.001
Never	47.39	45.62	51.37	
Former	34.95	31.91	28.35	
Current	17.66	22.47	20.29	
Race (%)				< 0.001
Mexican American	5.26	4.96	4.09	
Other Hispanic	5.38	5.62	5.87	
White	79.73	77.16	76.12	
Black	6.75	7.34	3.43	
Others	2.88	4.92	6.20	
Education levels (%)				< 0.001
Less than high school	24.29	19.58	17.90	
High school or equivalent	25.96	27.58	22.32	
College or above	49.75	52.85	59.78	
Ratio of family income to poverty	3.17 ± 1.64	3.27 ± 1.59	3.45 ± 1.56	< 0.001
Hypertension (%)	50.12	42.02	39.72	< 0.001
Diabetes (%)	13.12	11.19	9.17	0.003
SBP (mmHg)	131.23 ± 20.69	127.12 ± 18.80	125.55 ± 19.17	< 0.001
DBP (mmHg)	72.64 ± 14.68	73.81 ± 12.75	75.34 ± 11.20	< 0.001
TC (mmol/L)	5.43 ± 0.96	5.40 ± 0.99	5.37 ± 0.99	0.810
HDL (mmol/L)	1.35 ± 0.41	1.36 ± 0.41	1.39 ± 0.42	0.114
TG (mmol/L)	1.47 (1.03, 2.11)	1.40 (0.97, 2.08)	1.32(0.90, 1.96)	0.002
BUN (mmol/L)	5.53 ± 1.88	5.11 ± 1.67	5.10 ± 1.67	< 0.001
UA (mmol/L)	328.65 ± 87.47	319.35 ± 81.25	315.70 ± 86.57	< 0.001
e-GFR (ml/min/1.73m2)	87.80 ± 26.73	90.83 ± 25.22	93.91 ± 27.17	< 0.001

*Notes* BMI: body mass index, SBP: systolic blood pressure, DBP: diastolic blood pressure, TC: total cholesterol, HDL: high density lipoprotein, TG: triglyceride, BUN: blood urea nitrogen, UA: uric acid, e-GFR: estimated glomerular filtration rate

### Association between LTL and all-cause motality and CVD mortality

During a median follow-up period of 217 months, the weighted all-cause mortality rate was 28.58%, while the CVD mortality rate was 8.32%. We constructed multivariate weighted Cox proportional hazards regression models to assess the relationship between LTL and both all-cause and CVD mortality. The results are presented in Table 2. For each unit increase in the natural logarithm of the ratio of LTL to single-copy gene copy number (ln(t/s ratio)), there was a significant 51% decrease in the risk of all-cause mortality (HR: 0.49, 95% CI: 0.35–0.69, *P* < 0.001), and a 58% decrease in the risk of CVD mortality (HR: 0.42, 95% CI: 0.23–0.78, *P* = 0.006).

**Table 2:** Association between telomere length and mortality outcomes

	Model I	Model II	Model III
**All-cause mortality**			
ln (t/s ratio)	0.16 (0.12–0.22), < 0.001	0.40 (0.29–0.56) < 0.001	0.49 (0.35–0.69) < 0.001
Short tertile	1.00	1.00	1.00
Middle tertile	0.57 (0.49–0.66), < 0.001	0.76 (0.64–0.89) 0.001	0.80 (0.67–0.95) 0.009
Long tertile	0.39 (0.33–0.46), < 0.001	0.62 (0.52–0.75) < 0.001	0.65 (0.54–0.78) < 0.001
*P* for trend	< 0.001	< 0.001	< 0.001
**CVD mortality**			
ln (t/s ratio)	0.11 (0.06–0.19), < 0.001	0.33 (0.18–0.61) < 0.001	0.42 (0.23–0.78) 0.006
Short tertile	1.00	1.00	1.00
Middle tertile	0.54 (0.41–0.71), < 0.001	0.78 (0.57–1.06) 0.118	0.90 (0.65–1.24) 0.511
Long tertile	0.32 (0.23–0.44), < 0.001	0.57 (0.40–0.81) 0.002	0.64 (0.45–0.93) 0.018
*P* for trend	< 0.001	0.002	0.018

Data were presented as HR (95% CI) and *P*-value

Model I was crude model. Model II was adjusted for age, gender, smoking status, education levels, race, body mass index and ratio of family income to poverty, Model III was further adjusted for hypertension, diabetes, systolic blood pressure, diastolic blood pressure, blood urea nitrogen, total cholesterol, high density lipoprotein, triglyceride, uric acid and estimated glomerular filtration rate

**Fig. 1:**
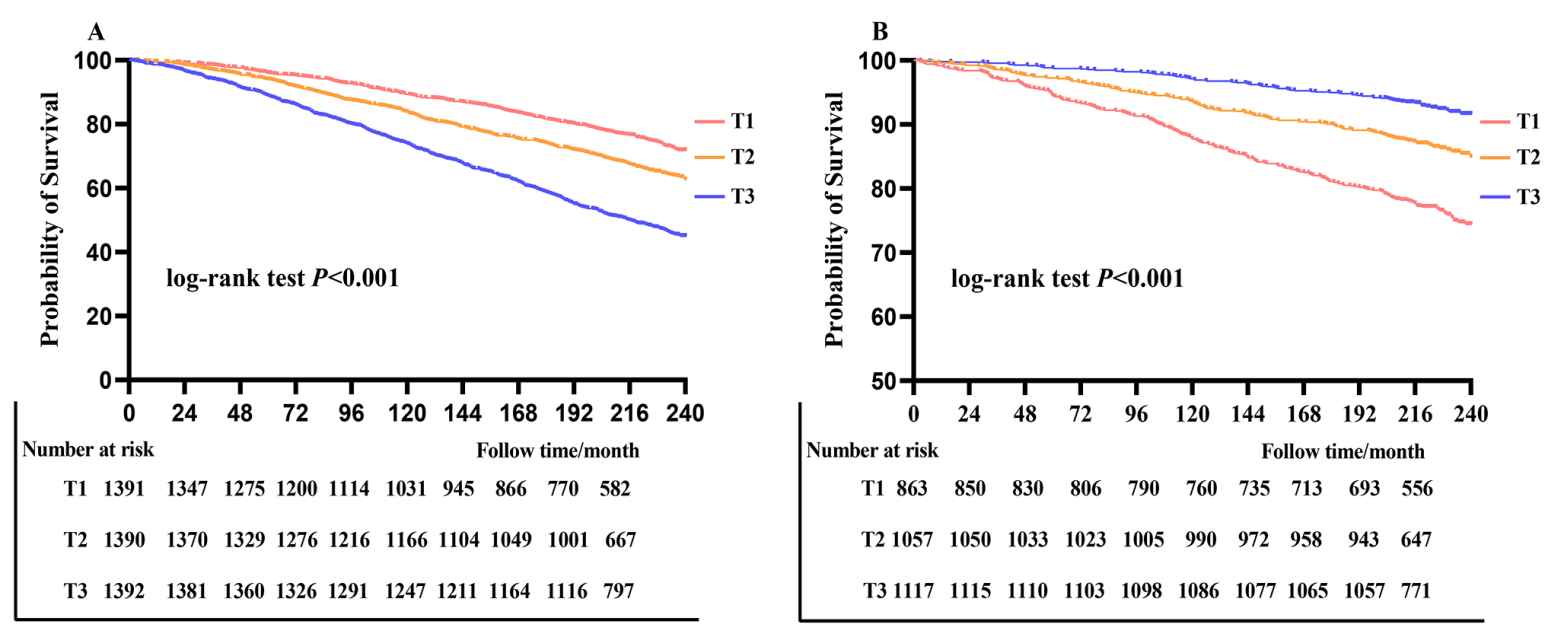
Kaplan-Meier curves for LTL associated with all-cause mortaltiy (**A**) and CVD mortality (**B**)

**Fig. 2:**
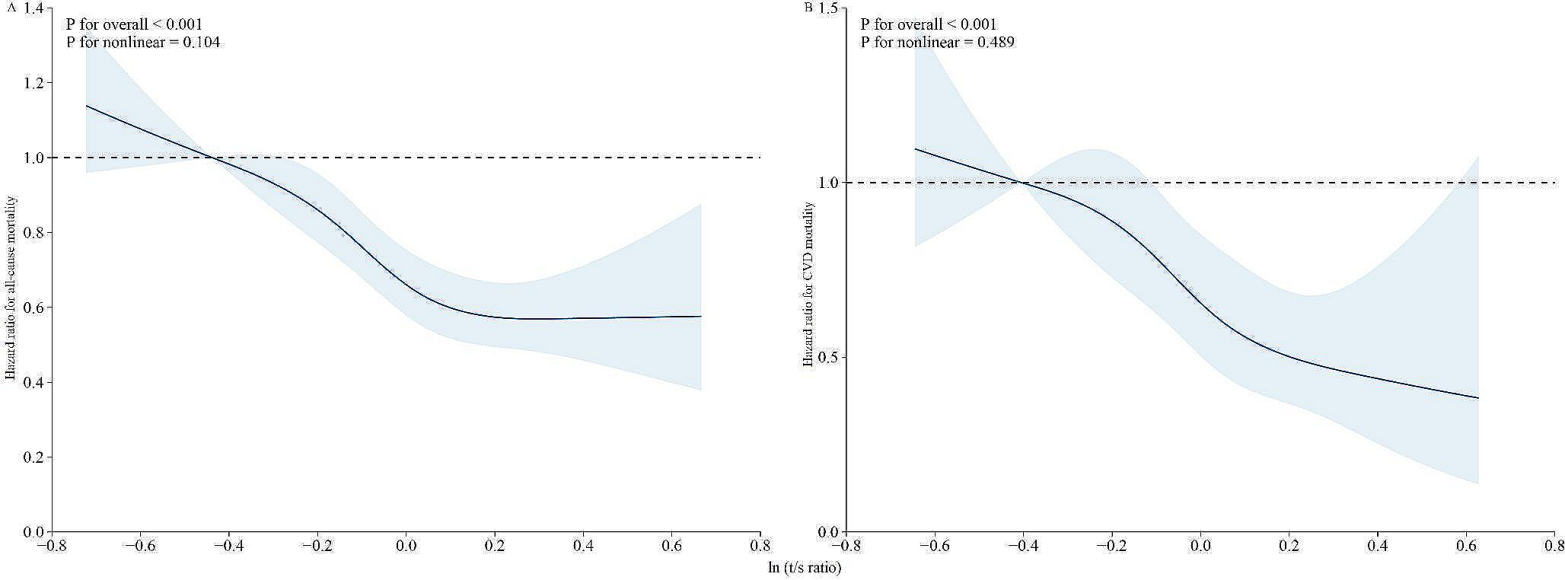
The non-linear dose-response association between LTL and all-cause mortality (**A**) and CVD mortality (**B**)

Furthermore, quantile analysis revealed a significantly decreased risk of all-cause (HR: 0.65, 95% CI: 0.54–0.78, *P* < 0.001) and CVD mortality (HR: 0.64, 95% CI: 0.45–0.93, *P* = 0.018) among participants in the T3 group compared to those in the T1 group. Kaplan-Meier survival curves demonstrated a significant association between low telomere length and increased risks of all-cause and CVD mortality (log-rank test *P* < 0.001) (Fig. 1). RCS curves indicated a non-linear dose-response association with all-cause mortality and CVD mortality (*P* for non-linearity of 0.106 and 0.489, respectively) (Fig. 2).

### Subgroup and sensitivity analysis

**Fig. 3:**
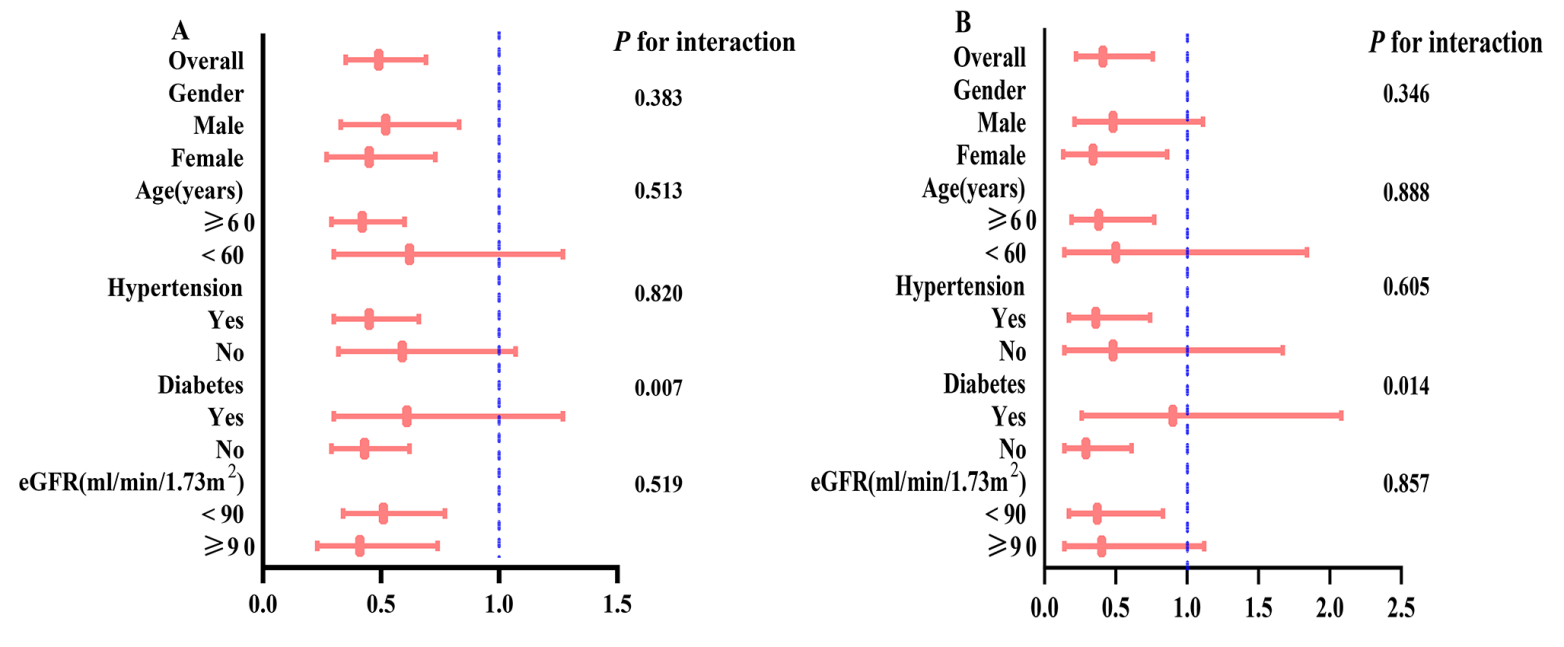
Subgroup analysis for the association between LTL and all-cause mortality (**A**) and CVD mortality (**B**)

Subgroup and sensitivity analyses were conducted to investigate the robustness of the association between LTL and mortality in middle-aged or older individuals without CVD. None of the examined variables, except for diabetes, demonstrated a significant modification of the relationship (*P* for interaction > 0.05) (Fig. 3). This suggests that individuals without diabetes who had longer LTL exhibited a significantly lower risk of all-cause and CVD mortality. Additionally, we performed sensitivity analysis by excluding individuals who died within the first 2 years of follow-up, and consistent results were obtained (Table 3).

**Table 3:** Sensitivity analysis for the association between telomere length and mortality outcomes

	Model I	Model II	Model III
**All-cause mortality**			
ln (t/s ratio)	0.17 (0.12–0.23), < 0.001	0.40 (0.29–0.56) < 0.001	0.49 (0.34–0.68) < 0.001
Short tertile	1.00	1.00	1.00
Middle tertile	0.58 (0.49–0.68), < 0.001	0.77 (0.65–0.91) 0.002	0.81 (0.68–0.96) 0.017
Long tertile	0.40 (0.34–0.47), < 0.001	0.64 (0.53–0.77) < 0.001	0.68 (0.56–0.82) < 0.001
*P* for trend	< 0.001	< 0.001	< 0.001
**CVD mortality**			
ln (t/s ratio)	0.11 (0.06–0.19), < 0.001	0.34 (0.18–0.63) 0.001	0.42 (0.22–0.79) 0.007
Short tertile	1.00	1.00	1.00
Middle tertile	0.54 (0.40–072), < 0.001	0.78 (0.57–1.07) 0.130	0.88 (0.64–1.23) 0.459
Long tertile	0.32 (0.23–0.44), < 0.001	0.57 (0.40–0.82) 0.002	0.64 (0.44–0.92) 0.016
*P* for trend	< 0.001	0.002	0.016

Data were presented as HR (95% CI) and *P*-value

Model I was crude model, Model II was adjusted for age, gender, smoking status, education levels, race, body mass index and ratio of family income to poverty, Model III was further adjusted for hypertension, diabetes, systolic blood pressure, diastolic blood pressure, blood urea nitrogen, total cholesterol, high density lipoprotein, triglyceride, uric acid and estimated glomerular filtration rate

## Discussion

To the best of our knowledge, this study is the first to explore the potential predictive value of LTL for long-term mortality in middle-aged or older individuals without CVD. The main findings of this study were as follows: (1) Longer LTL was independently associated with a reduced risk of all-cause and CVD mortality. (2) There was a linear dose-response relationship between LTL and the hazard ratio for mortality.

Middle-aged and older individuals are particularly susceptible to age-related diseases, including CVD, malignant tumors, and multi-organ dysfunction (MOD) [16, 17]. Consequently, the mortality rate in this specific population is significantly increased. Shortened TL, a biological marker of aging, has been shown to be significantly associated with an increased risk of all-cause mortality. A recently published prospective cohort study involving 2046 patients with pulmonary fibrosis demonstrated a negative association between LTL and chronological age (*R* = -0.28, *P* < 0.001), with shorter LTL serving as an independent predictor of all-cause mortality across racial backgrounds [18]. Chen et al. found that among participants with diabetes, each unit increase in LTL was significantly associated with a 47% decrease in the risk of death from any cause [19]. Another study, which included three longitudinal studies (Cardiovascular Health Study, Framingham Heart Study, and Women’s Health Initiative), revealed that a 1-kilobase decrease in LTL was significantly associated with a 34% increased risk of all-cause mortalit [20]. In our study, we also found a significant negative association between LTL and age (Pearson analysis: *r* = -0.32, *P* < 0.001). With long-term follow-up, shortened LTL was significantly associated with an increased risk of all-cause mortality among middle-aged and older individuals without pre-existing CVD.

In this study, the diagnosis criteria for the CVD was based on the self-reported by participants during interview, which was consistent with previous studies that based on the large-scale epidemiological data [21, 22]. Self-reported health data is valuable for large-scale epidemiological studies due to practicality and cost-effectiveness, inherently carry the risk of information bias. This limitation is particularly relevant in the context of cardiovascular conditions, where misreporting—whether due to recall bias, lack of medical diagnosis, or misunderstanding of the condition’s nature—can significantly influence the accuracy of the data collected. Recognizing this constraint, we adjusted our analyses for a wide range of covariates known to be associated with cardiovascular health and telomere length, including but not limited to lifestyle factors (smoking status), education levels, socioeconomic status, and comorbid conditions (hypertension and diabetes). This approach helps to partially control for the potential confounding effects of misclassification bias. Despite these efforts, the possibility remains that the self-reported nature of CVD status could dilute the observed associations between leukocyte telomere length and mortality risks. This concern underscores the need for future research utilizing more objective measures of cardiovascular health, such as clinical assessments or biomarkers, to validate and extend our findings. The impact of self-reported data on the interpretation of our results highlights a broader issue in epidemiological research—the balance between the breadth of data collection enabled by self-reporting and the depth and accuracy provided by clinical measures. Our study contributes to the ongoing dialogue on this topic by demonstrating both the potential and the limitations of self-reported health data in assessing the complex interplay between genetic markers, disease risk, and mortality.

CVD currently ranks among the leading causes of death worldwide, posing a significant burden on both the economic and public health systems [23, 24]. Aging, an unmodifiable factor, independently contributes to the development of CVD and CVD-associated mortality [25]. Despite cumulative evidence establishing a significant association between telomere length and CVD, the association between telomere length and CVD mortality remains controversial. In a recent prospective cohort study involving 1980 participants with metabolic syndrome, individuals with the shortest TL had a 41% increased risk of CVD mortality compared to those in the highest group, with a median follow-up period of 17.75 years [26]. Andrea N. et al. conducted an analysis of telomere-associated gene polymorphisms and found that women with OBFC1 genotypes associated with longer TL had a significantly decreased risk of CVD mortality, while such an association was not observed in men [27]. Ute Mons et.al [28] conducted a prospective study using two population-based cohorts (ESTHER study and Nurses’ Health Study) and found that TL was not a significant risk factor for long-term CVD mortality. A recently published study investigated the association between telomere length and mortality among patients with diabetes and found that TL was not significantly associated with death from any cause. The authors even found that longer TL was associated with an increased risk of CVD mortality, contradicting previous studies [12]. In our study, we examined the association between LTL and CVD mortality among middle-aged and older participants without prior CVD. We observed that participants in the longest LTL group had a 36% decreased risk of mortality. Additionally, we found that the association between LTL and all-cause mortality, as well as CVD mortality among participants with diabetes, was not significant in the subgroup analysis. Overall, the association between TL and all-cause mortality, as well as disease-specific mortality, remains a controversial issue, necessitating further studies.

One possible explanation for the observed association is that shortened TL is indicative of unhealthy aging. In this study, individuals with shorter TL were older, more likely to be male, and had significantly abnormal levels of physical examination and laboratory tests. These findings suggest that TL may serve as a predictor of unhealthy aging among middle-aged and older subjects without CVD. Moreover, cellular senescence has been proposed as a potential mechanism underlying the association between shorter TL and increased mortality risk. As telomeres become excessively shortened, cells enter a state of senescence, which involves the release of pro-inflammatory compounds and permanent cessation of cell division [29]. This process is believed to contribute to several age-related disorders, such as osteoarthritis, atherosclerosis, and frailty [30, 31].

Our study possesses several notable strengths, including a substantial sample size and comprehensive control of multiple confounding variables. Additionally, our study featured a prolonged follow-up duration, with a median follow-up time of 217 months, allowing us to assess persistent mortality risk. However, it is crucial to recognize that our research was observational in nature, precluding the establishment of causality. Furthermore, the diagnosis of CVD relied on self-reporting, which can introduce biases, including recall bias and misclassification due to participants’ misunderstanding of their health conditions, potentially leading to the inclusion of asymptomatic patients in the cohort and influencing the analysis. A significant limitation of our study was the evaluation of telomere length at a single time point, which may not capture potential fluctuations in telomere length over time. Moreover, the study population exclusively consisted of individuals from the United States, and therefore, the findings may not be generalizable to other populations with distinct demographics.

## Conclusion

Among middle-aged or older individuals without prior CVD, LTL may be applied as an important biomarker for the risk stratification of all-cause mortality and CVD mortaltiy in the long-term.

## Data Availability

The datasets used and analyzed during the current study are available from the corresponding author on reasonable request.
